# The neural bases of vitality forms

**DOI:** 10.1093/nsr/nwz187

**Published:** 2020-02-24

**Authors:** Giuseppe Di Cesare, Marzio Gerbella, Giacomo Rizzolatti

**Affiliations:** 1 Cognitive Architecture for Collaborative Technologies Unit, Italian Institute of Technology, Genova 16163, Italy; 2 Department of Medicine and Surgery, Neuroscience Unit, University of Parma, Parma 43125, Italy; 3 Istituto di Neuroscienze, Consiglio nazionale delle Ricerche, Parma 43125, Italy

**Keywords:** action observation, social interactions, emotions, insula, mirror mechanism

## Abstract

Unlike emotions, which are short-lasting events accompanied by viscero-motor responses, vitality forms are continuous internal states that modulate the motor behaviors of individuals and are devoid of the autonomic modifications that characterize real emotions. Despite the importance of vitality forms in social life, only recently have neurophysiological studies been devoted to this issue. The first part of this review describes fMRI experiments, showing that the dorso-central insula is activated during the execution, the perception and the imagination of arm actions endowed with different vitality forms as well as during the hearing and the production of speech conveying vitality forms. In the second part, we address the means by which the dorso-central insula modulates the networks for controlling action execution and how the sensory and interoceptive information is conveyed to this insular sector. Finally, we present behavioral data showing the importance of vitality forms in social interactions.

## EMOTIONS AND VITALITY FORMS

The *Book of Rites*, a Chinese encyclopedia from the first century, lists seven basic emotions, defining them as the *feelings of men.* These emotions were joy, anger, sadness, fear, love, disliking and liking. Many modern authors accept this notion of discrete basic emotions, although disagreement exists on their number and types [1–3].

Several authors consider emotions to be ‘action programs’ triggered by perceived or recalled external stimuli [1,2,4]. A great merit of Darwin was establishing the notion that each of these emotions corresponds to specific innate facial and bodily configurations that differ based on the quality of feeling. According to him, these innate models evolved as ‘signals’ that are understood by all members of a species to increase their survival probability.

Basic emotions are short-lasting events, although typically ending soon after their triggering stimuli cease. As stressed by James [5], viscero-motor responses accompany all emotions, which, according to him, represent the real essences of emotional states. Note that, after experiencing an emotion, an agent may think about the emotional context or emotional stimuli that caused it, and this emotionally driven cognitive state may persist in a human for a long time. However, these states cannot be considered emotions because they are devoid of the vegetative storms that characterize real emotions [5].

The definition of emotion discussed above allows one to differentiate emotion from another type of affective state that Stern [6] named *vitality affect*. Vitality affects, also called vitality forms [7], are internal states that modulate human motor behavior in a *continuous* manner and, unlike emotions, are not discrete. As Stern writes, the same action can be performed in different ways depending on the positive or negative attitudes that an individual has toward others. For example, a caress could be delicate or rushed and a handshake could be gentle or vigorous in the absence of any corresponding discrete emotion.

Vitality forms characterize social interactions by providing information about the affective states of the agents involved. Indeed, when interacting with another individual, the execution of a rude or gentle action enables one to communicate his or her mood. Conversely, the observation of these vitality forms allows an observer to understand the mood or attitude of an agent.

The ability to express and understand vitality forms are already present in infants, suggesting that they are primordial ways to relate to and understand others [6–10]. In the absence of vitality forms, all actions would be similar and devoid of any affective color. According to the information they provide, vitality forms are characterized by different kinematic properties: velocity, trajectory, energy and power [6,11]. Globally, these kinematic properties provide specific experiences to the observer that reflect the affective state of the agent.

Despite the crucial role of vitality forms in interpersonal relations, very little is known about their neurophysiological bases; only recently have data started to be collected on this issue. The aim of the present article is to review these data.

This article has four sections. In the *first* section, we present fMRI data obtained by presenting arm actions endowed with different vitality forms. These data show that the dorso-central insula is crucially involved in the perception of arm action vitality forms. Previous studies have indicated that the same sector is also activated when an agent *performs* actions endowed with vitality forms. The overlap of execution and perception of action conveying the vitality forms suggests that the dorso-central insula is endowed with the mirror mechanism. In the *second* section of the review, we present fMRI data showing that the same insular sector is also active during the presentation and the emission of speech conveying vitality forms. In the *third* section, we address the means by which the dorso-central insula may modulate the cortical networks for controlling action execution and how sensory and interoceptive information is conveyed to this insular sector. Toward this end, we present some tractography and connectivity data collected from both humans and macaque monkeys. Finally, in the *last* section, we discuss behavioral data showing the importance of vitality forms during social interactions.

## THE LOCALIZATION OF ACTION VITALITY FORMS IN THE INSULA

As discussed in the introduction, the term ‘vitality form’ describes *how* an action is performed, regardless of its goal. To localize the specific region activated when subjects focus their attention on action vitality forms, an fMRI study was performed [11] in which participants were presented with video clips showing interactions between two actors.

**Figure 1.**
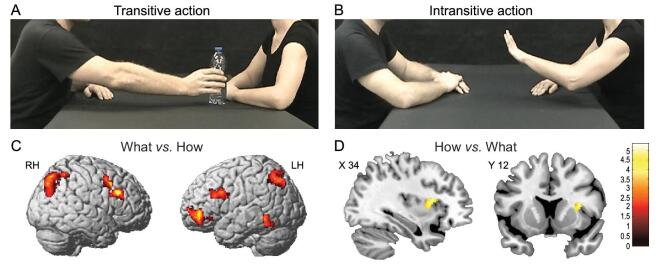
Example of video clips observed by participants showing an action performed with the object (passing a bottle) (A) or without the object (stop gesture) (B). Brain activations resulting from the contrast between the *what* task and *how* task (C) and the *how* task and *what* task (D), respectively. Figure adapted from [11].

The interactions comprised four transitive actions (grasping a cup, passing a bottle, giving a packet of crackers and passing a ball; Fig. 1A) and four intransitive actions (clapping hands, shaking hands, stroking the other actor's backhand and stopping gestures; Fig. 1B). Each action was performed with one of two different vitality forms (gentle and rude). The stimuli were presented in pairs of consecutive videos in which the observed action (*what*) and vitality (*how*) could be the same or could change between the video pairs. The participants also had to perform two tasks (*what* and *how*). In the *what* task, the participants were required to pay attention to the aims of the actions observed in the two consecutive videos and decide whether the two actions were the same or different, regardless of their vitality form. In the *how* task, the participants were required to focus their attention on the action vitality forms and to decide whether the vitality forms were the same or different in the two consecutive videos, regardless of the type of action performed.

In both tasks, activations were found in the parieto-frontal circuit classically involved in the observation and execution of actions with stronger activations for the *what* task, relative to the *how* task, in the ventral premotor cortex, in the posterior parietal lobe bilaterally and in the inferior frontal gyrus of the left hemisphere (Fig. 1C). The contrast of *how* and *what* revealed a specific activation in the right dorso-central insula (Fig. 1D). While the activation of the parieto-frontal circuit during action observation was an expected finding [12], the selective activation of the central part of the insula when participants focused on the action vitality forms was the first demonstration that this sector of the insula plays a specific role in processing vitality forms.

Actions expressing vitality forms are characterized by specific physical properties [6], among which velocity is the most salient. Therefore, the activation of the dorso-central insula during the observation of action vitality forms was possibly due to velocity coding rather that than to action vitality form coding. This problem was addressed by Di Cesare *et al.* [13] using multivoxel pattern analysis. The authors presented participants with video clips showing transitive actions (passing a bottle, a jar and a can) performed at three velocities (low, middle and high) and asked them to focus their attention either on the velocities of the actions or on their vitality forms. The results showed that the dorso-central insula contained discriminative voxels selectively tuned to vitality form processing.

In a subsequent fMRI study, Di Cesare *et al.* [14] more directly investigated the notion, derived from the previous experiment, that the dorso-central insula is involved in processing action vitality forms. In addition, they tested whether this insular region was also active during the *execution* of actions endowed with vitality forms.

**Figure 2.**
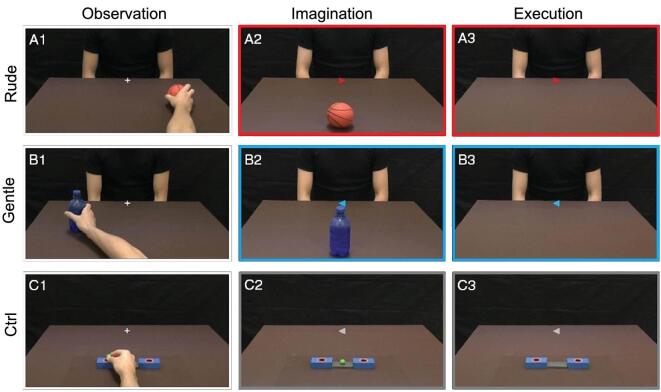
Experimental task design. Left panel: observation task. The participant observed the right hand of an actor moving an object in the rightward (A1) or leftward (B1) directions. The observed action was performed gently or rudely and the task required participants to focus on the style of action. As a control, some participants observed the right hand of the actor placing a ball in the right or left box (C1). Middle panel: imagination task. The participants were required to imagine themselves passing an object to another actor displayed in front of them with either a gentle or a rude vitality form. In the central part of the screen, a cue indicated the vitality forms (blue: gentle; red: rude) and the direction of the imagined action (A2 and B2). As a control, the participants had to imagine placing the ball in the box according to the direction of the cue (C2). Right panel: execution task. The participants held a package of crackers and had to move it with rude (A3, red color) or gentle (B3, blue color) vitality forms toward the actor displayed in front of him. As a control, the participants had to place the small ball in the box. Figure adapted from [14].

The experiment was carried out on 15 healthy, right-handed participants. The participants were required to perform three different tasks: observation (OBS), imagination (IMA) and execution (EXE). In the observation task, the participants observed video clips showing an actor passing an object to another one in either a gentle or a rude way (vitality condition; VF observation task) or an actor placing a small ball in a box (control condition; CT observation task). In the execution task, the participants were required to move an object in a rude or gentle way (VF execution task) or to place a small ball in a box in the most neutral way possible. Finally, in the imagination task, the participants were asked to imagine themselves passing an object toward the actor facing them in a gentle or in a rude way (VF imagination task) or to imagine placing a small ball in the box without any specific vitality form (CT imagination task) (Fig. 2).

**Figure 3.**
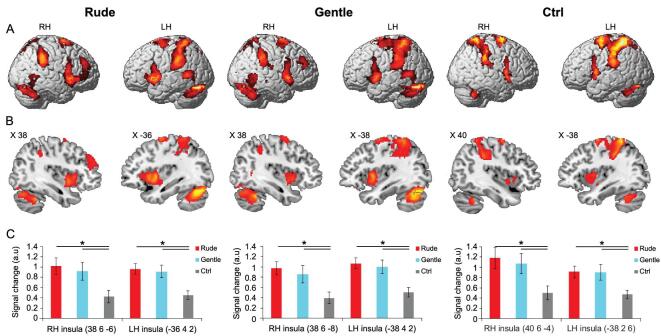
Overlapping areas active during the three different tasks (OBS, IMA and EXE). (A) Lateral views of the right and left hemispheres. The activations in the three conditions (rude, gentle and ctrl) were obtained with a conjunction analysis. (B) Parasagittal sections showing the insular activations in the three conditions. (C) Signal changes in six regions of interest created on the central insula. Asterisks indicate significant differences at *P* < 0.05, Bonferroni-corrected. Figure adapted from [14].

The results showed that, during OBS, there were bilateral activations of the occipital lobe and of the parieto-frontal circuits involved in processing hand and arm actions. Similar activation patterns were observed during the IMA task but with much weaker, less extended activations of the occipital areas. Finally, during EXE, activations were found in the same parieto-frontal circuits as in the other two tasks, as well as strong activations of the left somatosensory and motor cortices. Figure 3 shows the overlap of the areas activated in all three tasks (OBS, IMA and EXE).

For both conditions (gentle and rude) and in all three tasks, the conjunction analysis showed bilateral activations of the premotor and parietal cortices and strong activations of the left somatosensory cortex, the motor cortex and the dorsal part of the cerebellum (Fig. 3). Most importantly, the analysis also revealed, in all three tasks, strong activations of the dorso-central insula. These data indicate that, as sensory representations of the action goal are transformed into motor representations of the same goal in the parieto-frontal circuits (mirror mechanism), a similar mirror transformation also occurs in the dorso-central insula, transforming the visual representations of perceived vitality forms into their motor representations.

The finding that the dorso-central part of the insula is involved in vitality form processing fits with the general functional organization of the insula in monkeys and humans. Experiments with monkeys have shown that electrical stimulation of the dorso-central part of the insula elicits body-part movements with a rich representation of the movements of the upper limb [15]. These movements are radically different from the complex motor behaviors obtained by the stimulation of the rostral insula, the stimulation of which elicits complex positive ingestive behavior dorsally and negative ingestive behavior (i.e. disgust, vomiting) ventrally.

A similar organization pattern was reported by Kurth *et al.* in humans [16]. In a meta-analysis based on a very large number of functional neuroimaging studies, these authors found four distinct functional fields in the human insula: the sensorimotor, the socio-emotional, the olfactory–gustatory and the cognitive fields. The sensorimotor field corresponds to the analogous sensorimotor functional field of a monkey. It also corresponds to the insula sector involved in vitality form production and perception.

## SPEECH VITALITY FORMS

Vitality forms can be conveyed, not only through gestures and actions, but also through words. According to the attitude of the speaker toward the listener or his or her mood, the speaker talks gently or rudely. Thus, words conveying vitality forms enable the speaker to communicate his or her internal state and allow the listener to understand the speaker's mood.

The capacity to perceive speech vitality forms is already present in infants [6]. Indeed, during mother–child interactions, mothers often pronounce words using a characteristically childish language. Specifically, such mothers slow down the pronunciation of words, adapting their language to the perceptive capacities of their children [17,18].

An interesting question to clarify is whether the dorso-central insula, which is involved in the processing of action vitality forms, is also involved in encoding speech vitality forms. One fMRI study addressed this issue [19]. Sixteen right-handed participants were presented with auditory stimuli consisting of four Italian action verbs (‘dammi’ [give], ‘prendi’ [take], ‘tocca’ [touch] and ‘strappa’ [tear]) pronounced by two actors (a male and a female). All the action verbs were pronounced using two different vitality forms: rude and gentle (vitality condition). For each action verb, two controls were used: a ‘robotic’ voice pronouncing the same action verbs as the actors (robot condition) and a scrambled version of the four verbs (scrambled VF condition). The ‘robotic’ voice maintained the word meaning without conveying any vitality form. The scrambled stimuli controlled for the physical properties (pitch and amplitude) of the verbal stimuli without conveying the words’ meanings or vitality forms.

**Figure 4.**
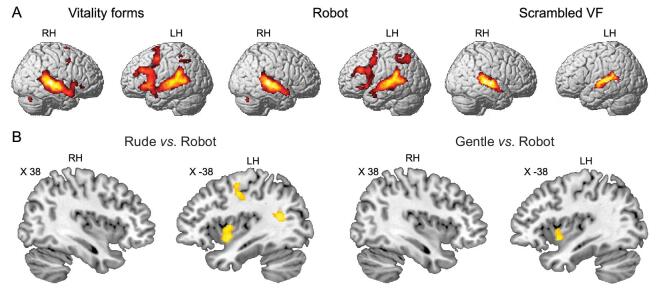
Brain activations obtained from hearing different stimuli categories (A). Parasagittal sections showing the activations resulting from the contrast of *rude vs. robot* and *gentle vs. robot* (B). Figure adapted from [19].

Figure 4A shows the brain activations under the vitality form, robot and scrambled conditions. Hearing vitality form action words produced activations of the superior temporal gyrus, left inferior parietal lobule, left premotor, left prefrontal cortex, posterior part of the inferior frontal gyrus and, most importantly, bilateral activation of the insula. A similar activation pattern was observed under the robot condition except for the insula, the activation of which was present only under the vitality condition (Fig. 4). Listening to the scrambled stimuli exclusively activated the auditory temporal areas. The direct contrasts of *vitality forms vs. robot* and *vitality forms vs. scrambled VF* showed, in all cases, significant activation of the left dorso-central insula (Fig. 4B).

The finding that the dorso-central insula was activated when participants listened to action verbs cannot simply be accounted for by the meaning of those verbs. Indeed, although the robotic voice conveyed exactly the same meaning, the insula was activated only when participants listened to action verbs conveying vitality forms. Similarly, the physical properties (intensity and frequency) of the action verbs could not have been responsible for the insula activation. In fact, the scrambled stimuli did not produce any activation of the insula even though their physical properties were the same as those of action verbs.

In a subsequent fMRI study, the same research group tried to establish whether the dorso-central insula involved in vitality form speech perception also becomes active during the imagery of action verbs internally generated with different vitality forms [20]. The experiment was based on the fact that, in the fMRI experiment, movements could not be studied for technical reasons and, therefore, the researchers could not directly investigate vitality form speech production. The authors thus used the motor imagery of the same action verbs previously used for the vitality form speech perception as a strategy for assessing the possible activation of the insular cortex during the *production* of vitality forms. Indeed, as shown by Jeannerod [21], motor imagery activates the same circuits that become active during action execution, with the exception of the primary motor cortex.

The experiment was carried out on 16 participants who were required to perform two tasks: listening (LST) and speech imagination (IMA). In the listening task (*VF listening*), the participants listened to three Italian action verbs (‘prendi’ [take], ‘tocca’ [touch] and ‘chiudi’ [close]) pronounced by two Italian actors (a male and a female) in gentle and rude ways (vitality condition). As a control (*CT listening*), the participants listened to the spellings of three nonsensical words (D-I-M-A, I-R-P-A and M-A-P-A) pronounced by the same actors. The spelled nonsensical words had the same physical properties of the vitality speech stimuli (pitch and amplitude) but did not convey any vitality form. In the speech imagination task, the participants were required to imagine pronouncing the same action verbs as the listening task in a rude or gentle way (*VF imagination*) or to imagine pronouncing the spelling of the three nonsensical words without any vitality forms (*CT imagination*).

**Figure 5.**
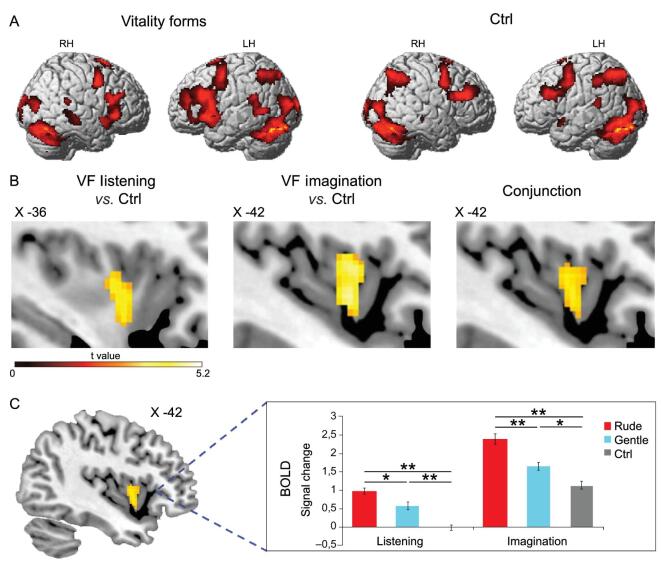
Brain activations obtained in speech processing. (A) Overlap of areas active during the listening (LST) and speech imagination tasks (IMA) obtained with a conjunction analysis for both vitality and ctrl conditions. (B) Parasagittal sections showing the left insular activations during the speech session in the contrast of vitality *vs*. ctrl during LST and IMA tasks. The conjunction analysis in the speech session revealed a common activation of the dorso-central sector of insula in the LST and IMA tasks (B, right panel). (C) Bold signal changes extracted from the left dorso-central insula resulting from the conjunction analysis of the speech tasks (LST and IMA). The horizontal line above the columns indicates the comparisons among the rude vitality form, the gentle vitality form and the control. The bars indicate the standard error of the mean. Asterisks indicate significant differences set at *P* < 0.05 (*) and *P* < 0.001 (**). Figure adapted from [20].

The results showed that listening to action verbs pronounced with gentle and rude vitality forms activated the parieto-frontal circuit related to action understanding, more strongly on the left side, along with activating the temporal superior frontal gyrus bilaterally. Imagining the pronunciation of the same action verbs with the same vitality forms produced a similar activation pattern, except for the superior temporal areas, and a stronger activation of the rostral prefrontal lobe. Figure 5A shows the overlap of the areas activated in both tasks (LST and IMA). Most importantly, in both tasks, there was activation, relative to controls, of the dorso-central insula. The conjunction analysis showed that the same insular sector was active in both tasks (Fig. 5B). Finally, it is interesting to note that, as for action vitality forms, the sensory representations of speech vitality forms are transformed in the dorso-central insula into corresponding vitality form motor representations. This indicates that the mirror mechanism is present in the dorso-central insula for speech as well.

## THE ANATOMICAL CONNECTIONS OF THE DORSO-CENTRAL INSULA

The results reviewed so far show that the dorso-central insula is involved in the expression and perception of the vitality forms of actions and speech. Because this sector of the insula has no direct connections with centers controlling arm and mouth movements, an important point to clarify is how the dorso-central insula may modulate an agent's actions. Considering the fundamental role of the parieto-frontal circuit in controlling voluntary arm and mouth actions [12], it is plausible that the dorso-central insula modulates agents’ expressions of mood and attitudes toward others throughout the activity of this circuit. Tract-tracing investigations carried out in monkeys support this possibility, as they have shown that the dorso-central insula is connected with all the three key nodes (the inferior parietal lobule [IPL], the ventral premotor cortex [PMv] and the prefrontal area 46) of the arm-and-hand control circuit [22–24].

**Figure 6.**
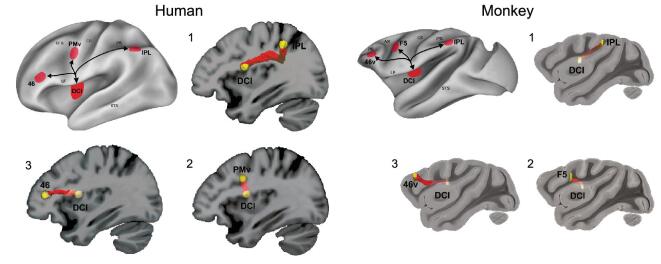
Insular connections to the parieto-frontal grasping circuit in humans and monkeys. Overview of insular white-matter tracts connecting the dorso-central insula to the parieto-frontal grasping circuit in humans (left side) and monkeys (right side). White-matter tracts connecting the insula to the inferior parietal lobe (1), the premotor cortex (2) and the prefrontal area (3). Figure adapted from [25].

A probabilistic tractography study (DTI) carried out with 15 right-handed participants illustrated that, as in monkeys, the human dorso-central insula is connected with all the aforementioned nodes of the parieto-frontal circuit [25]. In the same study, a DTI was carried out in four monkeys to compare the insula circuitry in the two species. The results showed that, in both humans and monkeys, the white-matter tracts connecting the dorso-central insula with the parieto-frontal circuit correspond to the third branch of the superior longitudinal fasciculus and the arcuate fasciculus, indicating that this insula-cortical network has been maintained throughout the evolution of primates. Figure 6 illustrates the anatomical fiber tracts.

The link of the dorso-central insula with the parieto-frontal circuit is consistent with a recent review of studies involving monkeys showing that this sector of the insula is also connected with other ‘sensorimotor’ cerebral territories [26], among which the arm-and-hand representation of the second somatosensory cortex [27], the hand sector of the skeletomotor putamen [28] and the middle part of the cingulate cortex [29], in which electrical stimulation occurred in both monkeys and humans, evoke arm movement [30–32]. These connections, on the one hand, confirm the involvement of the dorso-central insula in the cerebral networks for generating hand action and, on the other hand, suggest additional routes through which the insula can modulate the vitality forms of the agents.

Another important issue is to clarify the means by which, during interaction with other individuals, the visual and acoustic inputs reach the dorso-central insula, allowing one to recognize the vitality forms expressed by others. In monkeys, the central part of the insula receives connections from the anterior part of the superior temporal sulcus [33], hosting neurons responding to complex visual stimuli, including different types of biological hand and arm movements [34], and from the rostral auditory parabelt [35], which processes complex acoustic stimuli, such as conspecific calls [36]. Similarly, in humans, Almashaikhi *et al.* [37] showed that the dorso-central insula is functionally connected to temporal territories encoding visual and acoustic biological stimuli, such as the observation of hand movement and listening to voices [38]. These anatomical pathways represent the main routes through which visual and acoustic information reach the dorso-central insula.

**Figure 7.**
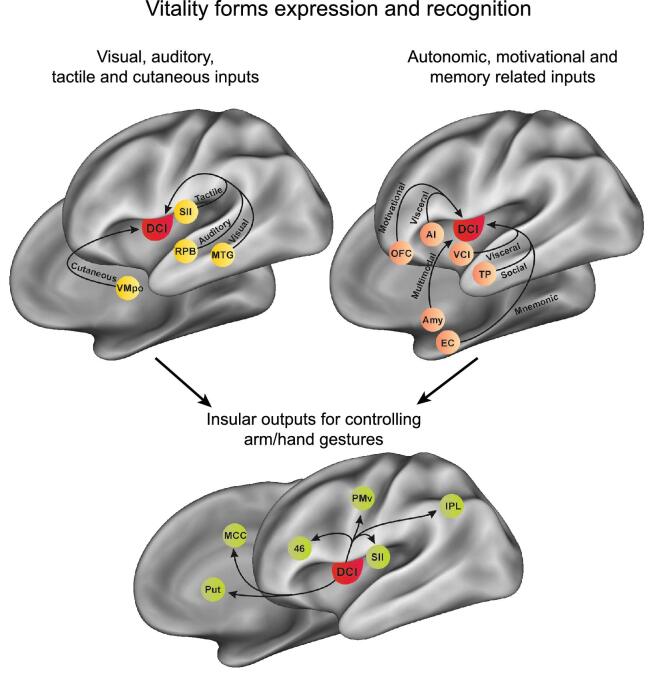
Model depicting the main anatomical pathways of the dorso-central insula and the hypothesized information flow, indicated by the arrows, during the recognition and the expression of vitality form. AI, anterior insula; Amy, amygdala; EC, entorhinal cortex; IPL, inferior parietal lobule; MCC, middle cingulate cortex; MTG, middle temporal gyrus; PMv, ventral premotor cortex; Put, putamen; OFC, orbitofrontal cortex; RPB, rostral parabelt; SII, second somatosensory cortex; TP, temporal pole; VCI, ventrocentral insula; VMpo, posterior part of the ventral medial nucleus.

The anatomical pathways described above allow one to define how the dorso-central insula modulates motor behavior and the means by which an individual can recognize the vitality form of another agent, but they do not describe how the interoceptive information concerning the affective state of an agent is conveyed to this insular sector. Concerning this issue, in a series of studies, Craig [39,40] showed that the dorso-central insula receives many types of interoceptive information from a specific thalamic nucleus, the ventromedial posterior (VMpo), which receives direct projections from lamina I spinal neurons and hosts cells responding to many types of cutaneous stimuli, such as pain, temperature sensations and affective touch (CT-fibers). Consistently, the dorso-central insula was shown to be activated by affective touch in an fMRI study on humans [41] and to contain neurons that encode dynamic touches perceived as pleasing in a monkey electrophysiology investigation [42]. These findings suggest that, when one feels pain or other cutaneous sensations, such as affective touch during interaction with others, the dorso-central insula may transform the affective state of an agent into the corresponding vitality form (Fig. 7).

In addition to receiving interoceptive afferences from the thalamus, the dorso-central insula is connected to some cortical territories involved in socio-emotional processes strongly linked with the autonomic nervous system and, therefore, can provide information on the affective state of an agent. In particular, the dorso-central insula receives projections from sectors of the adjacent anterior and ventral insula, which encode emotional and visceral states [40,43], as well as from cortical regions that integrate the emotional aspects of sensory stimuli with reward and memory, such as the temporal pole and orbitofrontal and entorhinal cortices [43–46] (Fig. 7). On the basis of these studies, perhaps when perceiving or recalling stimuli endowed with emotional or motivational content, the dorso-central insula transforms the evoked affective state into the corresponding vitality form (Fig. 7 shows this model in a pictorial form).

The descending subcortical connections of the dorso-central insula are limited to the skeletomotor putamen and to a weak input to the lateral nucleus of the amygdala [26]. It is interesting to note that, on the contrary, the adjacent ventrocentral and anterior parts of the insula are strongly connected to various subcortical structures, including the hypothalamus, the ventral tegmental area, the ventral striatum and almost all the nuclei of the amygdala [22] (see Fig. 7). Consistently with these subcortical connections, the electrical stimulation of the ventrocentral and the anterior insula evokes emotional behaviors, affiliative motor acts (e.g. lip-smacking) from the ventrocentral insula and disgust from the anterior insula. As described in the introduction, viscero-motor responses are essential components of emotions. Thus, if we assume that subcortical projections are necessary to have a ‘real’ emotional state, the paucity of the subcortical connection of the dorso-central insula explains why humans express and perceive vitality forms in the absence of the viscero-motor responses typical of basic emotions and stresses; once more, this constitutes the difference between vitality forms and emotions.

## THE ROLES OF VITALITY FORMS IN SOCIAL INTERACTIONS

**Figure 8.**
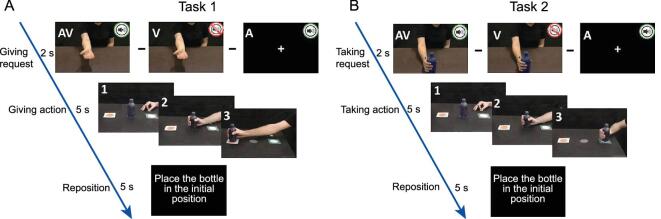
Experimental paradigm of the kinematic study. Participants were presented with audio-visual (AV), visual (V) and auditory (A) stimuli. In task 1, after the request, participants were requested to give the bottle (A). In task 2, after the request, the participants were requested to take the bottle (B). Panels with numbers display the phases of the participants’ movements during the experimental trial: 1, starting position; 2, grasping the bottle; and 3, taking (or giving) the bottle. The timeline reports the timing of different trial phases. Figure adapted from [47].

When an agent performs or pronounces actions or words gently or rudely, a receiver immediately understands whether that agent is in a calm, friendly mood or in a bad, negative one. It is intuitive, therefore, that vitality forms expressed by an agent may positively or negatively influence the behavior of a receiver. A demonstration of this influence, however, was missing in neuroscience literature. Recently, a kinematic study was carried out to investigate the presence of this influence [47]. Fourteen participants took part in the study. During the experiment, participants were presented with video clips showing an actor and actress making gestural or verbal requests to acquire an object (e.g. ‘give me the bottle’; task 1, ‘giving’, Fig. 8A) or to act on it (‘take the bottle’; task 2, ‘taking’, Fig. 8B). Each request was presented as visual action (V: visual modality), speech request (A: auditory modality) or both together (AV: audio-visual) (Fig. 8). All requests were expressed with rude and gentle vitality forms. After the actors’ requests (V, A and AV), participants performed the required actions (grasping a bottle with the goal to give or take it).

The results showed that, for both tasks (‘giving’ and ‘taking’), the speech and action vitality forms expressed by the actors influenced the kinematic parameters (velocity and trajectory) of the subsequent actions performed by the participants. In particular, concerning the reaching phase, vitality forms modulated the temporal (acceleration and velocity) and spatial parameters (trajectory) of the reach component, showing a wider trajectory and higher velocity in response to the rude requests than to the gentle ones. Additionally, concerning the grasping phase, the results showed a wider maximal finger aperture in response to the rude vitality form than the gentle one. Taken together, these data indicate that the vitality forms expressed by the actors influenced both the reach and grasp components of the motor actions performed by participants. A possible interpretation of these data is that the insula of the receiver encodes the vitality forms of speech and actions and automatically transforms them into a motor domain, in this way preparing the appropriate motor response.

Vitality forms represent a fundamental aspect of social communication that characterizes human interactions and behavioral studies have shown that the perception of vitality forms is impaired in individuals with social and communicative disorders, such as children with autism spectrum disorders (ASD) [48,49]. ASD is a condition characterized by repetitive, stereotyped interest and difficulties in social interaction and communication [50].

Hobson and Lee [48] carried out a pioneering study on the capacity to understand and imitate the action style (*how*) of an observed action among a group of ASD participants. More specifically, the authors instructed children and youths (9–18 years old) as well as typically developing individuals (TD) (control group) to observe actions performed with objects in different styles (e.g. running a wooden stick across the ridges to make a vibrating sound in a rapid and forceful manner or doing so more slowly and gently). Each action had two components: a *what* (e.g. running the wooden stick) and a *how* (e.g. the vigorous or gentle manner). While participants in the control group imitated all the aspects of the observed actions, children with ASD imitated only the contents of the movements (the *what*) but rarely their action styles (the *how*). Accordingly, Cook *et al.* [51] showed that ASD individuals performed movements with atypical kinematics that correlated with a bias toward perceiving biological motion as unnatural. This finding is consistent with the idea of Trevarthen and Delafield-Butt [52] that, in autism, the impairment of body gesture perception affects social understanding.

In a subsequent behavioral study, Rochat *et al.* [47] asked children and youths with ASD, as well as TD controls, to observe video clips of two actors performing transitive and intransitive actions (e.g. giving a mug and giving a high-five) in vigorous and gentle ways. Video clips were presented in pairs, some pairs differing in the type of action (*what* task) others in the vitality forms (*how* task), and participants were required to judge whether video clips differed or not. Results showed that participants with ASD, compared to TD controls, significantly differed in the *how* task, while no difference was found in the *what* task. Taken together, these findings highlight impairments in the perception of vitality forms in children and youth with ASD, providing a point of reflection for professionals and caregivers who interact with ASD children and, on the basis of this research, may wish to facilitate perceptions of these aspects of social communication.

## CONCLUSIONS AND FUTURE PERSPECTIVES

**Figure 9.**
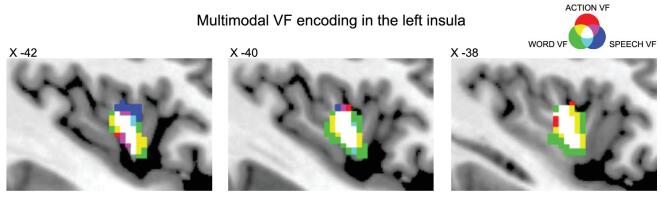
Multimodal encoding of vitality forms in the dorso-central insula. The image shows voxels activated during the processing of vitality forms in three fMRI experiments described in this review. Red color indicates voxels activated during the observation, imagination and execution of action performed with vitality forms relative to control conditions. Green color indicates voxel activated during the listening of action verbs pronounced with vitality forms relative to control stimuli (listening task). Blue color indicates voxels activated when participants listened action verbs (listening task) and imagined to pronounce them (imagination task) with vitality forms relative to control conditions. White color indicates voxels selective for vitality forms activated in all the three experiments.

In addition to the goals (*what*) and intentions (*why*) of actions, vitality forms constitute a third aspect of any human action (*how*) that is fundamental to interpersonal relations. The data reviewed in this study showed that the dorso-central insula represents the neural substrate of vitality forms (Fig. 9). This is true for the both the perception and expression of actions, indicating that this area is endowed with the mirror mechanism.

The future study of vitality forms may yield important discoveries. For instance, in the next few years, without a doubt, humans will increasingly interact with humanoid robots. A fascinating possibility is that new generations of robots will be endowed with capacities to express and comprehend vitality forms. Thanks to these capacities, robots may be able to detect the affective states of humans and, therefore, interact with them more effectively. Conversely, understanding robotic vitality forms could be fundamental to improving human–robot interactions [53]. Finally, these new programs installed in robots could also promote robot–robot interactions—an aspect that may not be secondary with an increased role of robots in our social lives.

The data reviewed in this article should be of interest to many scientists, including neuroscientists, robotics engineers, psychologists, child psychiatrists and researchers interested in social communication.
